# Observations on the Laws of Sympathetic Action in the Animal Œconomy

**Published:** 1833

**Authors:** James C. Finley


### WESTERN JOURNAL

OF THE

### MEDICAL AND PHYSICAL SCIENCES.

JANUARY, FEBRUARY AND MARCH, 1833.

### Essays and Cases.

### ART. I.—*Observations on the Laws of Sympathetic Action in the Animal Œconomy.* By JAMES C. FINLEY, M. D.

There has been a great deal of discussion, and much that we consider very unprofitable, among physicians, whether medicines operate by a sympathetic influence, or whether, being absorbed, they exert a direct influence upon the different parts of the body by being brought in contact with them through the course of the circulation.

It is not our intention to advocate, exclusively, either of these opinions. Foreign substances are undoubtedly taken into the circulation unchanged, and that, in this condition, they should exert their characteristic influence by a direct action upon the highly sensible surface of the vascular system, is no very improbable supposition, even if this opinion were not supported by the strongest circumstances, and upon the highest authority; but that their influence in this way is as general as the great body of the humoral pathologists suppose, we do not for a moment believe. But that they do not at all operate in this way however, is an observation that we should be far from hazarding, even in the absence of every thing like experiment upon the subject. The experiments of Majendie, if they could be implicitly relied upon, would settle the question that some poisons, the most virulent in their nature, and which, on account of the rapid development of their deleterious influence, would appear the most likely to act through the medium of the nervous system, produce their effect by absorption into the venous circulation. But, notwithstanding the high celebrity of Majendie, as an experimenter upon living animals, the great derangement of the animal functions which the subjects of his experiments must have experienced from the cruelty and severity of his operations, will render the inferences which he has drawn from them, the object to a very great proportion of the medical world.

Into this question we have no design to enter, but simply to enumerate some of the laws by which the different parts of the system exert a sympathetic influence that is productive of disease, or capable of being converted to salutary remedial agency. That such a connexion exists, is incontrovertible, from the instantaneous manner in which some medicines diffuse their influence over the system, as well as from the sympathy of the whole system with the *state* or *condition* of a particular organ, and in which consequently the nervous system, or the vascular system, *independent of the transmission of morbid agents*, must be the only medium of communication.

The precise limits of these two modes of influence can never be satisfactorily decided, not even with an approach to certainty. Probability and conjecture, in the great majority of cases, is the most that we can ever arrive at.

The different modes of sympathetic influence may be classed in the following manner: 1. The sympathy which connects the different parts of the body, and by virtue of which, the state or condition of any one member is transmitted throughout the whole system of organs, of which the body is composed. 2. The sympathy of Function. 3. The sympathy of structure.

We have a striking exemplification of this general bond of union which unites the different organs of the body in the phenomena which accompany either general or local inflammation. Inflammatory action induced in any organ of the body causes the system at large to sympathise in this same mode of action, to an extent, proportionate to the violence of the inflammation and to the importance or to the size of the organ; and, on the contrary, a state of general inflammatory action predisposes every part of the system, upon the occurrence of any local or accidental irritation, to take on a state of inflammation. Hence after severe injuries, we have not only a general phlogistic diathesis, but a liability to inflammation of the brain, concussion of the brain, or operations upon distant and delicate parts of the system, produce abscess of the liver, &c. Upon the same principle, we often witness in habits that are broken up by intemperance, a diseased action of the liver accompanied by an eruption upon the surface of the body, denoting, indeed, a morbid condition of the skin, but indicating at the same time, on the part of nature, an attempt to counteract the debility into which the viscera, connected with digestion, are running, by establishing a degree of inflammatory action in which the system sympathises. When, from general debility, or from the accidental application of any debilitating agent, the eruption recedes from the surface, depraved digestion, spasms of the stomach, with other indications of visceral derangement, and even sudden death, shew that the same want of action pervades the digestive organs. The same state of things is to be observed when the depression of the system in the stage of debility which precedes the eruption of the exanthemata, is such, either from general debility or from the application of cold, that the eruption does not take place, the serious local congestions indicate that this condition prevails in the whole system of internal organs as well as in the cutaneous structure.

The same identity of condition we find in the morbid effects produced by cold. When the application of cold is carried beyond the powers of reaction in the animal œconomy, we witness a paleness and constriction of the skin, and a deficient action of the capillary system on the surface of the body. Combined with these effects, and corresponding to them, we have a debilitated action of the heart and a corresponding derangement of capillary circulation pervading the capillary structure of the lungs, of the alimentary canal, and of the muscular and fibrous systems. That this is the general condition of the capillary system under these circumstances, we infer, from the diminished faculty of generating animal heat, from the diminution of the exhalations and secretions, from the visceral congestions, muscular cramps, and pain in the bones and ligaments, which result immediately from the application of cold; all which are followed by the occurrence of internal inflammations analogous to those which take place in the external parts of the body from the depressing effects arising from the direct application of cold.

A state of the system analagous to this, we find in the cold stage of intermittent fevers, where we find the cutaneous circulation almost arrested, the stomach affected with nausea and vomiting, the action of the lungs impaired, the viscera congested, and the brain and the nervous system in a state of torpor and insensibility; all resulting as analogous effects from a similar and wide spread derangement of the different organs of the animal œconomy; a derangement that is marked by defective vascular action, diminished secretions, impaired action of the heart, and venous congestion, effects of a common cause, and all acting and reacting upon each other.

Mistaken views of pathology have not unfrequently led pathologists to reject this simple view of the actions of the animal œconomy. Thus we hear of the derangement of the balance of the circulation and of the blood, and, consequently, of the powers of vitality being driven from one part to accumulate in another. Others, we hear speak of the revulsive power of remedial agents, as if the excessive action of one organ could be relieved by diverting that action to another. Profuse discharges from the bowels are again said to be produced by turning the current of the circulating fluids inward upon the bowels, and to be relieved by diverting them to the surface.

We do not intend to denounce that practice, by the appropriation of which, these theories are supported. But the theory is false, and the practice, if regulated by it, would be pernicious in the extreme. Views of this kind, therefore, as they serve only to give flippant routine practitioners a plausible hypothesis to satisfy the inquisitive public with a shew of knowledge, ought to be discountenanced by every intelligent practitioner.

Congestion and inflammation are as directly opposite in their character as are the stage of depression and reaction in intermittent fever; and this distinction when distinctly marked, pervades every tissue in the body. They may, however, exist together, because the latter is the remedy provided by nature against the injurious tendency of the former. When Dr. Rush defined the depressed state of the system which accompanies malignant fever as a state of suppressed excitement," or as a condition in which the system was so overpowered by excessive action that the usual systems of inflammation did not exhibit themselves, he advanced a doctrine which would be literally true, if the weakened pulse and the general prostration of the system depended, as in the case of inflammation of the stomach, upon the derangement of an organ whose healthy condition is essential to the vitality of the whole system. If, however, this was a condition in which full reaction was prevented by the serious nature of the congestions, and the great derangement of the capillary circulation, the theory was erroneous, and liable, in practice, to be perverted to a most pernicious influence.

This principle of general sympathy affords us an easy solution of the apparently diversified influence of local stimulants—blisters, rubefacients, &c. We see these applications, under different circumstances, successfully applied to remove inflammation, to relieve spasms of the muscles or viscera, to restore action to the surface, to obviate congestion, or to support the sinking powers of life. Do they effect these diversified indications by different principles of action adapted to the emergency, or by a uniform mode of action? By a uniform mode of action undoubtedly. Their first effect is to give tone to the capillary system, and through this, by an extension of their influence, to increase the action of the heart.

Rubefacients exert a decided influence upon the capillary system throughout the whole body, which, although less permanent than that produced by blisters, is more diffusive and hence better adapted to the cold stage of fevers and the temporary congestions which accompany them—indeed, to every form of capillary derangement which has not acquired a permanent character. They preserve much of this mode of action when taken into the stomach, and appear, when administered in this manner, to increase the action of the heart, rather by an indirect influence through the medium of the capillary circulation, than by directly increasing its action.

That both rubefacients and blisters do act in this manner upon the system, is evident from the fact, that they cannot be applied with advantage during the existence of a phlogistic diathesis. When, however, general action has been reduced, and inflammation is kept up by the inability of the weakened capillaries to throw off the load with which they are oppressed, or when inflammation, in its incipient stage, consists rather in an engorgement of the capillary vessels which has not, as yet, induced a similar condition of the blood vessels generally, then only, it is that local irritants relieve internal inflammation. This they do, not by a revulsive, but a directly stimulating property—not by inviting the fluids to a different tissue, but by imparting to the capillaries sufficient tone to enable them to restore their circulation.

The same principle of reasoning will apply to diseases of the bowels. In inflammations of their serous texture, all the powers of life are depressed—not because the powers of vitality are concentrated in the organs which are the seat of inflammation, but because the destruction of their functions prostrates the whole system. In dysentery or diarrhœa there is no other evidence of a change in the distribution of the fluids than the profuse discharges which are the common effect of a certain grade of inflammation in the mucous and serous membranes.

We have another exemplification of this general sympathy in the action of various medicines. Many of the humoral pathologists have labored very strenuously to prove that all these medicines operate only when taken into the circulating system. This opinion, however, cannot be for a moment entertained, and consequently the controversy must be respecting the lines of demarcation between those which operate upon the nervous system and those which produce their influence through the medium of the general circulation. Prussic acid, for instance, when applied to the extreme verge of vitality, may produce immediate death. Tobacco, when taken into the mouth, instantly produces universal muscular relaxation. Peruvian bark excites the action of the heart and arteries before it can possibly undergo the process of digestion.

In addition to these incontrovertible facts, the identity of operation which different medicines exhibit when applied to the different parts of the system, affords a strong proof that many medicines which are probably taken into the blood, do, nevertheless, produce their influence by a sympathetic influence. Tartar emetic, for instance, when received into the stomach, doubtless operates in virtue of the sympathy between the mucous and muscular coats of the stomach. When applied to the skin, however, or injected into the veins, it produces the same effect upon the muscular fibres. It cannot here be supposed that this result is produced by its reaching the stomach in the course of the circulation, for the act of vomiting is produced in conformity with a principle which we shall illustrate presently, by the irritation of the mucous membrane. The object of vomiting is to expel these substances from the stomach before they have an opportunity of being taken into the circulation, or of injuring the system in any other way, and besides, the peculiar relaxing influence produced by the mere contact of emetics with the mucous surfaces proves that they produce their specific effect by an impression upon the mucous surface of the stomach. The fact, therefore, that they produce the same effect when applied to a distant part of the body, may be adduced as another instance of the general sympathy which unites the different parts of the animal œconomy.

Another exemplification of this general law we have in the debility and fatigue which pervades all the organs of the body when any individual organ has been exhausted by overaction. This general relaxation is more strikingly exhibited when the debilitated organ is of great importance in the system; the stomach, for instance, the organs of generation, &c., although the influence is observable when any, even of the least important of the organs, are tasked beyond their power.

These are some of the most important instances of that law in virtue of which the whole system sympathises with each of its members, and takes on a general action analagous to that of the suffering organ. There are some apparent exceptions to this principle. To some of these we have already alluded. Another instance is to be found in the fact that the action of the skin and the kidneys is frequently in an inverse ratio. Of this fact the explanation is to be found in the circumstance that, as organs of excretion, the function of these is in several respects the same. If, therefore, the action of the one is increased or diminished, by a cause not sufficiently powerful to affect the system generally, the action of the other must be suspended or increased in a corresponding degree. A certain amount of the fluids in a healthy condition of the body are to be thrown off, and if they pass by one set of emunctories, they cannot of course by another. Of their general coincidence of action, however, we have abundant proof in a variety of diseases, as in fevers, dropsies, &c., in which both are partially suspended; and on the contrary, in the sweating stage and in the crisis of fevers, where both are increased. The same sympathy prevails between the liver and the skin, and, as a general principle, between all the tissues of the body.

The extent to which different organs sympathise with each other depends upon a variety of circumstances. The organs highly important, either on account of their being indispensable to vitality, or from their subservience to animal or social purposes, sympathise very readily with each other. The sympathy between the stomach and the brain is very intimate. The brain also sympathises very intimately with certain affections of the lungs, although the lungs generally do not exert that influence over the rest of the system which their importance would lead us to anticipate. The sympathies of the organs of generation are perhaps more decidedly marked than those of any other organ, for, besides the powerful influence they exert upon the health when the seat of disease, or wasted by debauchery; they, by an imperceptible influence mould the conformation of the body, and impress upon the mind the characteristics by which the sexes are distinguished.

Contiguity of position, also, has a very powerful influence in modifying sympathies; and this, even when there is no immediate connection by nerves or blood vessels—as, for instance, is the case between the thoracic and abdominal viscera and the integuments of those parts. The manner in which this intercourse between the different parts of the body is preserved, it is not easy to point out. It is generally attributed to the nervous system, and, perhaps in the majority of cases, with propriety. There are, however, doubtless, numerous other cases in which very powerful and decided sympathetic effects are communicated through the medium of the other great systems of the body, especially through the medium of the circulating system, by means of continuous sympathy, or by what may be called sympathy of function. The nervous system is doubtless essential to this extension of action, as it is to all the actions of vitality. It is, however, necessary only as essential to vital action of every kind.

*Sympathy of Function.* One of the most important and powerful of the bonds of union between the different parts of the body is derived from the close sympathy which exists between parts that are engaged in the same function; and between the instinctive passions and appetites, and the wants and sufferings of the organs to which they are subservient.

The close sympathy which subsists between the different parts of the circulating system we have already alluded to in speaking of general sympathy. There is, however a different kind of sympathy depending upon the relation in which they stand towards each other.

The blood vessels are supplied with their circulating fluids through the medium of the absorbents; and hence, as a general principle, the action of these two systems are in an inverse ratio. Withdraw a large portion of the blood by venesection, or permit an unusual quantity to flow off by any of the emunctories, and the absorbent vessels seize with avidity upon every thing that comes within their reach. Deprive an animal for any length of time of food, and thereby preclude the supply by which the blood is renovated, and the absorbents from the different parts of the body, which, under ordinary circumstances, convey but a small quantity of colourless lymph, are seen to be abundantly loaded with a high colored fluid, evincing that they are striving, by an extraordinary exertion, to supply the demands of the blood vessels.

The same sympathy we observe between the appetites and the functions to which they are subservient. We have a curious instance of this in the sympathy between the sensations of thirst, for instance, and the exhaustion of the fluids. A more remarkable and inexplicable fact could scarcely be selected as illustrative of the sympathy of function, there being not a single physiological fact to shew why the mucous surfaces of the throat and fauces should be associated with the deficiency of fluids in the vascular system, except the tendency which this location of the sense of thirst has to lead to the instinctive desire for liquids to supply the deficiency. A similar remark may be made with respect to the sensation of hunger—all the explanations which have been offered in explanation of this appetite being not only inadequate, but in the highest degree irrational.

A similar sympathy we observe between the breasts of the female and the organs of generation. These organs arrive at maturity about the same time; at a proper period during gestation, a new action takes place in the mammæ to prepare them for providing the nourishment of the offspring; and when gestation is suspended, either by parturition or the death of the fœtus, the secretion of milk soon follows. This sympathy is as marked in the diseases to which these organs are subject—the mammæ, especially, exhibiting symptoms of disease whenever the uterus is the seat of structural or functional derangement. It has been very fashionable to account for this sympathy by means of a nervous or vascular connection; but, although we might possibly admit of this mode of explanation in relation to these particular organs, in what manner could we make it apply to other sexual sympathies? How would we account, for instance, for the sudden growth and the rapid maturity of the physical organs and the intellectual faculties, which take place at the approach of puberty? a change which is undoubtedly owing to the influence of the sexual organs.

We have other exemplifications of this functional sympathy, in the manner in which every organ responds to the irritation of those parts of the system to which its action is subservient. Thus the introduction of food into the mouth provokes the action of the salivary glands; the passing of the food into the duodenum is followed by a flow of bile into that intestine; and certain medicines, when taken into the stomach, produce vomiting or catharsis—the muscular coat of these viscera being called into action by the irritation of their mucous surfaces. The correspondence between the irritation of the retina, by the rays of light, and the introduction of the iris, affords a beautiful example of this sympathetic action.

It may be laid down as a general principle, of which the recollection of every reader will afford him many illustrations, that there is a close connection between the action of a gland and the irritation of its excretory duct. The proper stimulus of every gland is in the irritation of its excretory duct, nor can a single instance be adduced in which its secretions are increased by a stimulus applied directly to its substance. In some cases the sensibilities of the gland appear to centre in the excretory duct. This is peculiarly the case in irritation of the mucous coat of the bladder, the pain of which is universally felt in the extremity of the penis.

Another instance of this sympathy we have in the increased action of the urinary organs after the introduction of saline medicines into the circulation. The kidneys are the outlet through which substances of this kind escape from the system; and by a sympathy between the blood vessels and the organ whose office it is to relieve them, an increased action takes place and the salts are carried off with a copious discharge of the watery parts of the blood. The general explanation is, that this increased secretion is produced by the direct stimulus of these salts upon the secreting vessels. An explanation, however, which would refer the increased action of the kidneys to an impression upon those parts of the system to which they are subservient, would be more in accordance with other analogies in the animal œconomy.

It will be observed that the function of many of these organs is so indispensably connected with the system at large, that the *sympathy of function* forms a general bond of union between the different parts of the animal œconomy. This is the case with the alimentary canal, which, on account of its agency in the nourishment of the system, has a *functional sympathy* with the whole vascular system, both of waste and supply, as well as with each individual organ. Hence the number and extent of its sympathies and the great sensibility with which it is consequently endued, render it a most convenient and efficacious medium for affecting the rest of the system in the administration of medicine.

The sympathies of the vascular system are equally extensive, and, if it were as conveniently within our reach, would afford a means of affecting the system, in the use of our remedies, more powerful than the alimentary canal.

The functional sympathies of the nervous system are, of course as extensive as sensibility itself. This system must indeed be the connecting link by which the different parts of the body are bound together. In some cases, functional sympathy can evidently be traced to a peculiar distribution of nerves; as is the case with all those parts of the body to which the nerves of respiration are distributed. Probably, as we become acquainted with the *final cause* of other arrangements of the nervous system, we will discover a similar provision for the other sympathies of animal and organic life; but at present, the different speculations which have been advanced upon this subject, are supported by little more than fancy and conjecture.

The third kind of sympathy is that which demands upon *similarity of structure*. We observe this kind of sympathy in the tendency of similar structures throughout the body, to take on a similar state of diseased action. Thus, when a rheumatic or gouty diathesis prevails in the system, we see the disease, at one time, simultaneously prevailing in different parts of the body; and at another, suddenly leaving one part and attacking another —thereby shewing that the predisposition prevails universally throughout the fibrous and ligamentary structure. This form of sympathy is most frequently observed in continuous structures. Thus we see some forms of disease pervade the whole surface of the alimentary canal; the mucous surfaces of the air passages, or of the urinary organs. Certain cutaneous diseases at once affect the surface of the body, and the internal surface of the lungs, while, in other cases, a similar sympathy seems to prevail between the surface of the body and the alimentary canal.

This species of sympathy seems to exist in a very striking degree between the different parts of the absorbent system—in virtue of which a very slight irritation at their extremities frequently produces serious inflammation of the glands. It has been very common to attribute this affection of the glands to the absorption of acrimonious matter. But the extension of the inflammation along the whole course of the absorbent until it arrives at the gland, renders it more probable that it is extended by continuous sympathy. The reason why the absorbents are more prone to be affected in this manner than the other parts of the vascular system is, probably, to be sought for in the fact, that in consequence of the double function which they perform, of *absorption* and of *assimilation*, it is necessary for them to be endued with a greater degree of sensibility, as well as with a closer sympathy between the different parts which compose this system. A similar sympathy, however, exists in the blood vessels, especially, in the venous system. A fatal inflammation, extending along the course of the veins, has frequently followed venesection; and is so liable to occur after the application of a ligature to the larger veins, as almost to preclude surgeons from performing an operation which once promised to prove of very extensive utility.

These are some of the bonds of union by which this complicated animal machine, consisting of an infinite variety of parts, and constructed for as great a diversity of purposes, is combined into a harmonious system, and animated by a common personal identity, to share in the burdens and participate in the pleasures of each individual organ of which its structure is composed.

